# *Helicobacter pylori* Urease: Potential Contributions to Alzheimer’s Disease

**DOI:** 10.3390/ijms23063091

**Published:** 2022-03-13

**Authors:** Augusto F. Uberti, Natalia Callai-Silva, Matheus V. C. Grahl, Angela R. Piovesan, Eduarda G. Nachtigall, Cristiane R. G. Furini, Celia Regina Carlini

**Affiliations:** 1 Laboratory of Neurotoxins, Brain Institute of Rio Grande do Sul (BRAINS) and Graduate Program in Medicine and Health Sciences, Pontifícia Universidade Católica do Rio Grande do Sul (PUCRS), Porto Alegre CEP 90610-000, RS, Brazil; afuberti@gmail.com (A.F.U.); nati.callai@gmail.com (N.C.-S.); matheusgrahl@hotmail.com (M.V.C.G.); 2 Center of Biotechnology, Graduate Program in Cellular and Molecular Biology, Universidade Federal do Rio Grande do Sul (UFRGS), Porto Alegre CEP 91501-970, RS, Brazil; angelapiovesan@gmail.com; 3 Laboratory of Cognition and Memory Neurobiology, Brain Institute of Rio Grande do Sul (BRAINS) and Graduate Program in Biomedical Gerontology, Pontifical Catholic University of Rio Grande do Sul (PUCRS), Porto Alegre CEP 90610-000, RS, Brazil; eduarda.godfried@gmail.com (E.G.N.); cristiane.furini@pucrs.br (C.R.G.F.)

**Keywords:** *Helicobacter pylori*, urease, neuroinflammation, tau hyperphosphorylation, pro-inflammatory cytokines, object recognition test, elevated plus maze, SH-SY5Y neuroblastoma cells, BV-2 microglia

## Abstract

Alzheimer’s disease (AD) causes dementia and memory loss in the elderly. Deposits of beta-amyloid peptide and hyperphosphorylated tau protein are present in a brain with AD. A filtrate of *Helicobacter pylori’s* culture was previously found to induce hyperphosphorylation of tau in vivo, suggesting that bacterial exotoxins could permeate the blood–brain barrier and directly induce tau’s phosphorylation. *H. pylori*, which infects ~60% of the world population and causes gastritis and gastric cancer, produces a pro-inflammatory urease (HPU). Here, the neurotoxic potential of HPU was investigated in cultured cells and in rats. SH-SY5Y neuroblastoma cells exposed to HPU (50–300 nM) produced reactive oxygen species (ROS) and had an increased [Ca^2+^]i. HPU-treated BV-2 microglial cells produced ROS, cytokines IL-1β and TNF-α, and showed reduced viability. Rats received daily i.p., HPU (5 µg) for 7 days. Hyperphosphorylation of tau at Ser199, Thr205 and Ser396 sites, with no alterations in total tau or GSK-3β levels, and overexpression of Iba1, a marker of microglial activation, were seen in hippocampal homogenates. HPU was not detected in the brain homogenates. Behavioral tests were performed to assess cognitive impairments. Our findings support previous data suggesting an association between infection by *H. pylori* and tauopathies such as AD, possibly mediated by its urease.

## 1. Introduction

*Helicobacter pylori* is a gastric pathogen known to infect approximately 60% of the world population, and in 2014 around 4.4 billion people were infected by this bacterium [1,2]. In underdeveloped countries prevalence of 80% has been reported [3]. *H. pylori* infection is associated with several gastric pathologies, such as chronic gastritis, peptic and duodenal ulcers, gastric adenocarcinoma and lymphoma of the mucosa-associated lymphoid tissue [4]. Epidemiological studies correlate *H. pylori* infection with various extra gastrointestinal pathologies [5,6], such as ischemic heart disease [7], glaucoma [8], cerebrovascular diseases [9], autoimmune pancreatitis [10], and neurodegenerative diseases such as Alzheimer’s disease (AD) [11,12], mild cognitive impairment [13] and Parkinson’s disease [14,15]. Specifically, in the case of AD, the correlation is based on the high incidence of *H. pylori* in these patients [12,16], as well as by the presence of anti-*H. pylori* antibodies in their cerebrospinal fluid [11,17]. In addition, Wang et al., in 2015 [18], reported that an *H. pylori* culture filtrate, obtained without lysis of the bacterium, induced hyperphosphorylation of tau in the hippocampi of rats, marked by increased phosphorylation in three specific tau sites, Thr205, Thr231 and Ser404. The *H. pylori* filtrate also caused activation of the GSK-3β kinase and tau hyperphosphorylation in mouse neuroblastoma cells (N2a). However, this filtrate did not induce a significant cerebral inflammatory response. Based on these data, the authors suggested that *H. pylori*-induced tauopathy could be induced by to exotoxin(s) produced by the bacterium, capable of penetrating the blood–brain barrier (BBB) and directly inducing tau phosphorylation. As the authors did not characterize the filtrate, nor attempted to isolate any of its components, the existence and/or identity of these exotoxin(s) remains unknown.

*H. pylori*’s urease (HPU) is a cytoplasmic protein that can also be found on the surface of the bacteria, presumably adsorbed by viable cells as a result of the lysis of bacterial subpopulations sensitive to stomach acidity [19]. HPU is the most abundant protein produced by *H. pylori*, allowing the bacteria to survive in the gastric mucosa due to the enzymatically generated ammonia that forms a neutral microclimate in the extremely acidic environment of the stomach [20]. Besides regulating the intracellular pH by releasing ammonia, HPU also alkalinizes the extracellular pH, as a result of the diffusion of ammonia to the periplasm and the environment around the bacteria [21]. Urease-negative *H. pylori* mutants are unable to colonize the stomach of gnotobiotic piglets and nude mice [22,23]. HPU is widely used for the diagnosis of *H. pylori* infection [24,25].

Previous studies by our group have shown that different ureases, including HPU, display biological properties independent of their enzymatic action, which can potentially contribute to the pathogenesis of infections caused by the urease-producing bacteria (for review see [26,27]). In platelets, HPU induces secretion and degranulation of ADP-containing granules, which leads to platelet aggregation, responses accompanied by the production of eicosanoids derived from the 12-lipoxygenase pathway [28]. Platelet activation, mediated by HPU, turns these cells into a pro-inflammatory phenotype [29], reinforcing the importance of neutrophil and platelet-mediated inflammation in the inflammatory process caused by *H. pylori* [30,31]. HPU also activates other cell types, such as monocytes, neutrophils, endothelial cells and gastric epithelial cells. Human neutrophils activated in vitro by HPU are protected from apoptosis and produce reactive oxygen species (ROS), and in vivo, HPU induces neutrophil migration in the mouse paw edema model [30]. Our group has shown that HPU participates in pro-angiogenic processes, suggesting that the urease may be involved in the development of gastric carcinoma [32]. Moreover, HPU weakens cell junctions [33], altering the integrity of the endothelial cell barrier and increasing paracellular permeability [34]. HPU also promotes the differentiation of human endothelial cells. This mechanism is related to the production of ROS, which together with the weakening of the endothelial barrier, supposedly contributes to the progression of *H. pylori* infection to gastric carcinogenesis [34]. 

Neuroinflammation has emerged as an important causal mechanism of dementia, especially in cases of Alzheimer’s disease (AD), which is the most prevalent type of dementia. A high degree of glial cell activation was observed in the early stages of the disease, suggesting a vulnerability of the CNS to inflammation in AD. As the disease progresses, extensive stunted brain tissue is triggered [35]. In humans, a study using positron emission tomography showed that very high peripheral levels of C-reactive protein, an easily measurable inflammation marker, are associated with increased microglial activation [36], thus confirming that peripheral inflammation can promote changes in the brain’s immune system. Therefore, chronic peripheral inflammation, such as that caused by chronic *H. pylori* infection, can contribute to the development of brain inflammation and neurodegenerative diseases [37]. Taking into consideration the epidemiological association between *H. pylori* infection and AD [1], the data showing that the *H. pylori* culture’s filtrate induces hyperphosphorylation of tau [18] and the fact that HPU is the most abundant virulence factor of the bacteria, it is important to investigate whether it could have a relevant role in the development of tauopathies. Considering HPU’s pro-inflammatory activity, its presence in microvesicles produced by the bacterium [38], as well as a free protein released into the bloodstream from inflamed gastric tissue, HPU could potentially contribute to disrupting the blood–brain barrier and thus reach the central nervous system, where it could exert neurotoxic effects. In this work, we aimed to evaluate the neurotoxicity of HPU on CNS cells in in vitro models. We also evaluated the in vivo effects of HPU on hippocampal homogenates, seeking to understand the signaling triggered by the urease. Behavioral experiments were performed to investigate a possible role of HPU in early AD symptoms.

## 2. Results

### 2.1. In Vitro Effects of HPU in CNS Derived Cell Lines

#### 2.1.1. Toxicity to Human Neuroblastoma Cells

**Figure 1:**
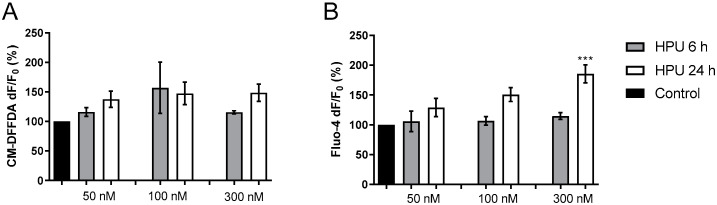
Toxicity of HPU to SH-SY5Y cells. (**A**). Undifferentiated SH-SY5Y cells were incubated with a CM-DFFDA (2 mM) probe for 30 min, 37 °C, in the dark. Cells were treated for 6 h (gray columns) and 24 h (white columns) with 20 mM NaPB (control) or 50, 100 or 300 nM HPU. Fluorescence was measured at λ_ex_ 495 nm/λ_em_ 527 nm. The results are expressed as a percentage of the control ± SEM and compared by one-way ANOVA followed by the Tukey test. (**B**). For calcium measurement, undifferentiated SH-SY5Y cells were incubated with a Fluo-4AM probe (5 μM, in 20 mM NaPB) for 45 min, 37 °C, in the dark. The treatment with 50, 100 or 300 nM HPU lasted for 6 h (gray columns) or 24 h (white columns). Controls with 20 mM NaPB (6 and 24 h) were considered 100%. Fluorescence was measured at λ_ex_ 488 nm/λ_em_ 530 nm. The results were expressed as a percentage of the control ± SEM and compared by one-way ANOVA followed by the Tukey test. *** *p* < 0.001 vs. control.

Undifferentiated human neuroblastoma cells SH-SY5Y were pre-loaded with the reactive oxygen species (ROS) fluorogenic probe CM-DFFDA (2 mM) and then treated with different concentrations of HPU (50, 100 and 300 nM). A tendency to increased production in HPU-treated cells was seen either after 6 or 24 h of exposition to the urease. On the other hand, SH-SY5Y cells showed significantly increased levels of intracellular Ca^2+^ upon 24 h of incubation with 300 nM HPU compared to control cells (Figure 1).

#### 2.1.2. Activation of BV-2 Microglial Cells

**Figure 2:**
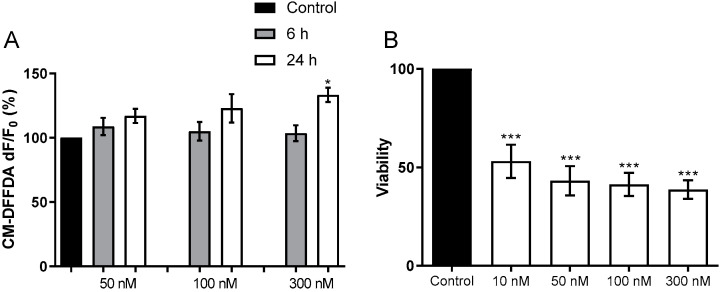
Toxicity of HPU to BV-2 cells (**A**) For ROS detection, BV-2 cells were incubated CM-DFFDA (2 mM) probe for 30 min, 37 °C, in the dark. After washing, BV-2 cells were incubated with 20 mM NaPB (control), or 50, 100 or 300 nM HPU for 6 h (gray columns) or 24 h (white columns). Fluorescence was measured at λ_ex_495 nm/λ_em_527 nm. The results were expressed as a percentage of the control ± SEM and compared by one-way ANOVA followed by the Tukey test. * *p* < 0.05 vs. controls. (**B**) BV-2 viability was analyzed by the MTT test after 24 h of exposure to HPU. The cultures’ supernatants were removed after the treatments and cells were incubated with MTT (5 mg/mL) for 4 h at 37 °C, then suspended in 100 µL DMSO. Absorbances were read at 570 nm. Mean ± SEM. *** *p* < 0.001 vs. control.

Microglia are the main cells of the brain’s innate immune system, displaying macrophage-like characteristics. Microglial activation into an M1 phenotype leads to an increase in pro-inflammatory cytokines, which contributes to neurotoxicity [39]. Our data showed that HPU-activated BV-2 cells produced ROS at the highest tested dose, and showed a marked reduction in viability after 24 h of treatment (Figure 2).

**Figure 3:**
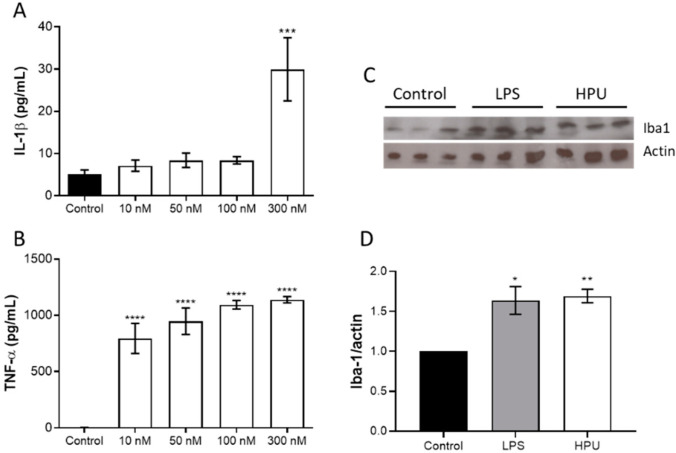
Microglial activation by HPU. For detection of cytokine expression, BV-2 cells were incubated for 6 h with HPU, and cultures’ supernatants were collected for detection of IL-1β panel (**A**) and TNF-α panel (**B**) by ELISA. The data (mean ± SEM) were analyzed by one-way parametric ANOVA with a Dunnett post-test. *** *p* < 0.001, **** *p* < 0.0001 vs. control. Panels (**C**,**D**): Iba1 levels in brain homogenates of HPU-treated rats were analyzed by Western blots (**C**), and quantified by densitometry (**D**). The data (mean ± SEM) were analyzed by one-way ANOVA followed by Bonferroni post hoc test. * *p* < 0.05, ** *p* < 0.01 vs. controls. All data were normalized against the endogenous actin content.

HPU also induced IL-1β and TNF-α production in BV-2 cells, indicating a pro-inflammatory phenotype (Figure 3).

### 2.2. Evaluation of Total and Phosphorylated Hippocampal Tau Levels in Rats Treated with HPU

**Figure 4:**
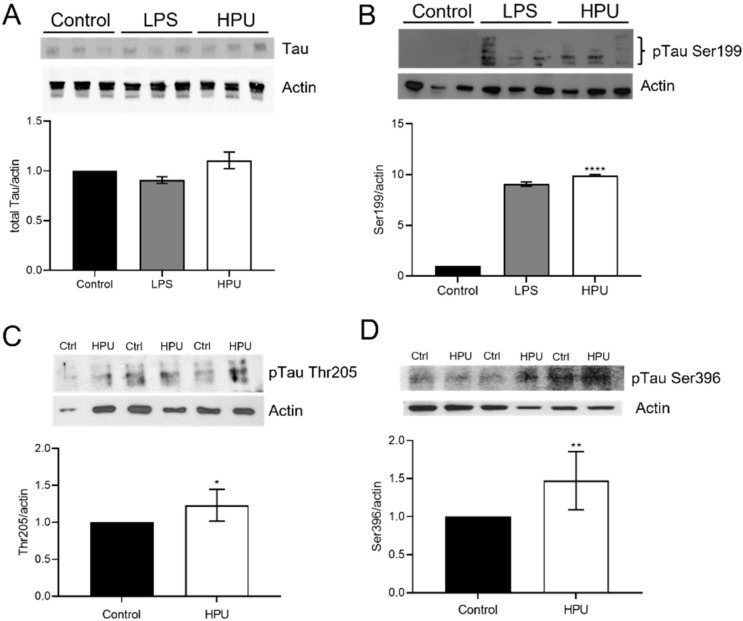
Quantification of total and phosphorylated tau protein in hippocampal homogenates of HPU-treated rats. Male 30-day-old Wistar rats received i.p. injections of HPU (5 µg/rat/day), LPS (1 mg/kg/rat/day), or sterile PBS for 7 days. Western blot assays were performed with hippocampal homogenates of animals from three independent experiments, each composed of groups with 4 animals each. Tissue brain homogenates were analyzed for total, panel (**A**) and phosphorylated tau at sites Ser199, panel (**B**), Thr205, panel (**C**) and Ser396, panel (**D**). The levels of tau protein were normalized by that of actin. The figure depicts representative blots and their densitometric analysis. Data are expressed as mean ± SD and analyzed by one-way ANOVA followed by Bonferroni post hoc test. * *p* < 0.05, ** *p* < 0.01, **** *p* < 0.0001 vs. controls.

Considering the data reported by Wang et al., 2015 [18] on tau protein phosphorylation in rats treated i.p., with *H. pylori* filtrates, in this work we sought to investigate if HPU could play a role in this process. In order to verify the effects of intraperitoneally administered HPU on rat hippocampal homogenates, Western blot assays were performed to evaluate microglial activation (Figure 3, panels C,D) and hyperphosphorylation of tau (Figure 4). Rats treated i.p., with HPU had an increased Iba1 content in the hippocampi, in levels similar to that seen in animals that received LPS treatment (positive control), consistent with the neuroinflammatory effect of the urease. 

The total content of tau protein was measured using a monoclonal anti-tau antibody (Figure 4A). No significant difference of total tau levels between the control and treated groups (HPU = 1.106 ± 0.082; LPS = 0.908 ± 0.034; normalized data) was seen. This result corroborates that of Wang et al., 2015 [18], who described no changes in the total tau levels in rats treated with *H. pylori* filtrate. On the other hand, in vivo treatment of rats with HPU promoted tau phosphorylation on Ser199, Thr205 and Ser396 sites (Figure 4B–D). Tau’s Ser199 phosphorylation site has been related to the early stages of AD development [40]. In this work, we found that the 7-day treatment with HPU promoted a 9-fold increase (*p* = 0.0001) of tau phosphorylation at the S199 site (9.93 ± 0.01), similar to that of the LPS-treated group (9.079 ± 0.209) when compared to the controls (normalized data, Figure 4B). A significant increase in phosphorylation levels in Thr205 of at least one of the three isoforms of tau was found in animals treated intraperitoneally with HPU for 7 days (1.233 ± 0.0847; *p* = 0.0249). The presence of tau phosphorylated at Ser396 was reported to be one of the first phosphorylation events in the early stages of AD development [41], detected even before conformational changes in the protein occur. Here an increase in tau Ser396 phosphorylation was seen in animals treated for 1 week with HPU (1.474 ± 0.1514; *p* = 0.014) compared to the control group.

### 2.3. Evaluation of GSK-3β Levels

**Figure 5:**
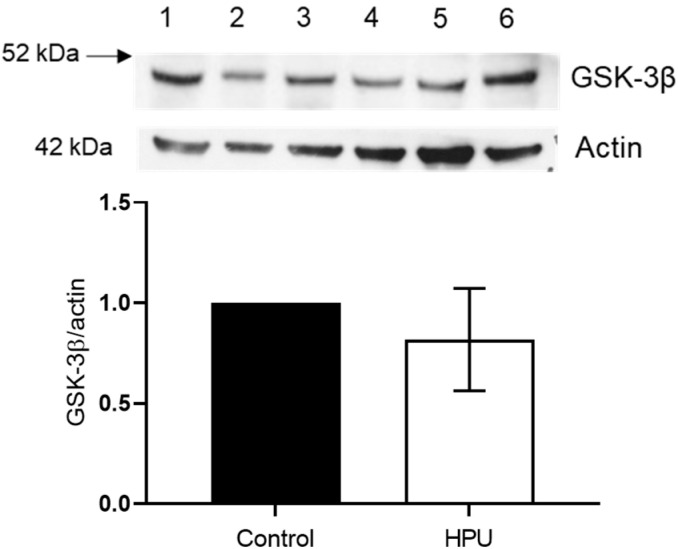
Quantification of GSK-3β kinase levels. Male Wistar rats received i.p. injections of HPU for 7 days (5 µg/rat/day), and the same volume of sterile saline was administered to the control group. Western blotting assays were performed with hippocampal homogenates of animals from three independent experiments (G1, G2, G3), each composed of a treated group and a control group (*n* = 4). Controls (lanes 1, 3 and 5) and HPU-treated (lanes 2, 4 and 6) homogenates were analyzed for GSK-3β and actin protein levels. The figure shows a representative blot and its densitometric analysis. Data are expressed as mean ± SD and analyzed by one-way ANOVA followed by Bonferroni post hoc test.

GSK-3β is an isoform of the GSK3 kinase abundantly found in the nervous system [42,43] and it phosphorylates tau sites usually related to the development of AD [44,45]. We investigated the levels of GSK-3β and no significant difference was found between the control group and HPU-treated rats (*p* = 0.2845) (Figure 5).

### 2.4. Blood–Brain Barrier Permeability of Rats Treated with HPU

**Figure 6:**
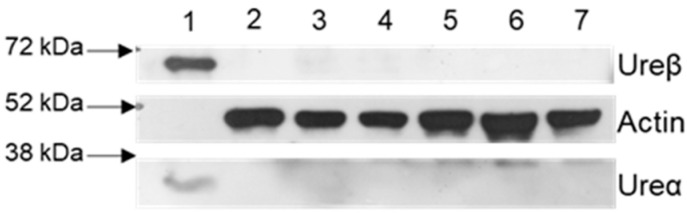
Blood–brain barrier integrity of rats treated with HPU. Male Wistar rats received i.p. injections of HPU for 7 days (5 µg/rat/day), and the same volume of sterile saline was administered to the control groups. Hippocampal homogenates from three independent experiments (G1, G2, G3), each composed of a treated group and a control group (N = 4), were analyzed by Western blot assays using antibodies against HPU subunit B (ureβ) and subunit A (urea) (60 kDa and 30 kDa, respectively). The figure illustrates a typical blot, with controls in lanes 2, 4 and 6, and HPU-treated groups shown in lanes 3, 5 and 7. Purified HPU protein (25 µg) is seen in lane 1.

To assess the permeability of the blood–brain barrier (BBB) to HPU, brain homogenates of the animals were analyzed by Western blot, using the primary antibodies anti-ureβ and anti-ureα, specific for detection of the urease’s B chain and A chain, respectively. As shown in Figure 6, the amount of HPU (if present) in the brain tissue of the treated rats is below the detection level (0.116 mg/mL). This result suggests that there was no damage to the integrity of the BBB under the tested conditions. 

### 2.5. Behavioral Tests

**Figure 7:**
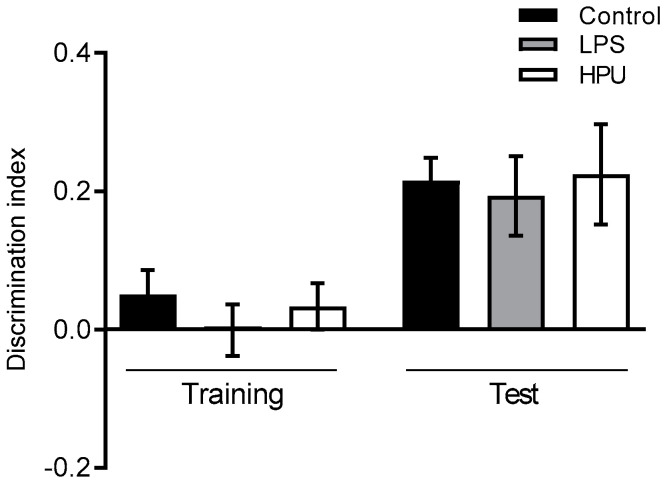
Effect of HPU on memory consolidation. Male Wistar rats received i.p., injections of HPU (5 µg/rat/day), LPS (1 mg/kg/rat/day, positive control) or saline (negative control) for 7 days. Discrimination indexes in the test phase were analyzed by one-way ANOVA followed by Bonferroni’s multiple comparison test. Data expressed as median ± SEM. *n* = 10 rats/group.

To investigate a possible role of HPU in early AD symptoms of *H. pylori*-infected patients, we evaluated memory and anxiety-like behavior in young adult Wistar rats. The object recognition test is used to test learning and memory, as rodents are innately curious and tend to explore novelty [46]. Rats without memory impairment would spend more time exploring the novel object in the test session. In our 7-day experiment, no difference was observed between groups, despite the biochemical features of AD being observed in the brains of HPU-treated rats, such as neuroinflammation and tau phosphorylation (Figure 7).

**Figure 8:**
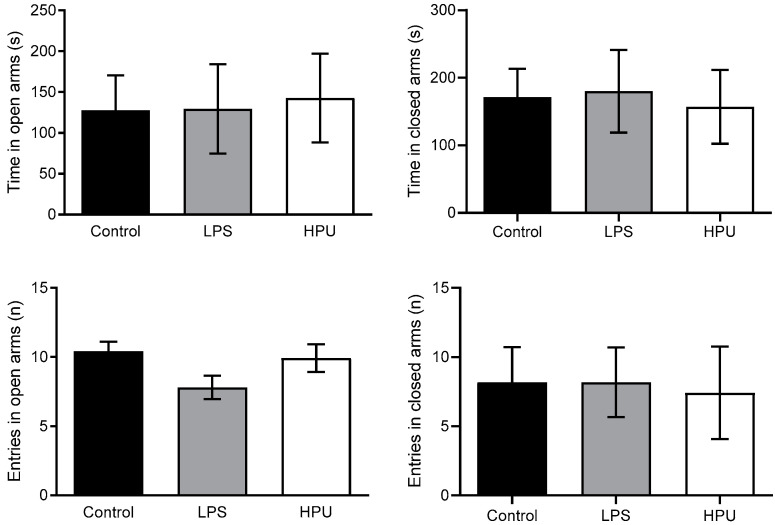
Elevated plus maze performance of HPU-treated rats. Male Wistar rats received i.p., injections of HPU (5 µg/rat/day), LPS (1 mg/kg/rat/day, positive control) or saline (negative control) for 7 days. Entries to open and closed arms of the elevated maze and time spent in each arm were measured. Data were analyzed by one-way ANOVA followed by Bonferroni’s multiple comparison test. Data expressed as median ± SEM. *n* = 10 rats/group.

The elevated plus maze test evaluates anxiety-like behavior, as rodents are naturally averted to open and elevated spaces [47]. In the 7-day experiment, all rats behaved similarly, with no signs of anxiety or discomfort, and no difference was observed among the groups (Figure 8).

## 3. Discussion

In 2009, Lo et al. [48] reported in vitro toxicity in gastric epithelial, neuroblastoma and microglia cell lines after treatment with the supernatant of an *H. pylori* culture medium, either directly or in co-culture. In addition, it was observed that the death of SH-SY5Y cells depended on the activation of BV-2 cells, suggesting that microglia play an important role in *H. pylori*-induced neurotoxicity. Considering that HPU represents 10 to 15% of the protein content of *H. pylori* [19,27], this urease is certainly present in the culture’s supernatant, which these authors called *H. pylori*-conditioned medium.

Physiological concentrations of ROS in epithelial cells can act as signals for several biological responses, including regulation of adhesion molecules and chemokines, formation of intercellular spaces and leukocyte migration [34,49,50]. Our group has previously demonstrated that HPU induces a significant increase in ROS production in neutrophils [30] and HMEC-1 endothelial cells [34]. In this work, we observed a tendency toward elevated ROS levels in HPU-treated SH-SY5Y and a significant increase of ROS production in BV-2 cells after 24 h of treatment. The same dose range of HPU which produced other effects described here was used in the ROS experiment. It is possible that ROS production follows distinct kinetics in SH-SY5Y cells and production could be greater in other experimental times. Moreover, the production of ROS could be extracellular and thus not detected by the CM-DFFDA probe.

The calcium ion (Ca^2+^) is a second intracellular messenger that regulates different cellular functions, such as membrane excitability, exocytosis, synaptic activity and gene expression [51]. Ca^2+^ in neurons is the coupling factor between excitation and propagation of electrochemical signals, intervening in many neuronal physiological responses to chemical and electrical stimuli. Changes in the levels of intracellular Ca^2+^ can also cause neuron death, and its homeostasis is carefully controlled [52]. In neurons, the concentration of Ca^2+^ is maintained at very low levels, and an exacerbated increase in cytosolic [Ca^2+^] can result in neuronal death by necrosis, while a prolonged decrease in cytosolic [Ca^2+^] leads to the induction of cell death by apoptosis [53,54]. In SH-SY5Y cells, which have a neuronal origin, HPU promoted cytosolic [Ca^2+^] increase in a dose and time-dependent manner. An increase in cytosolic [Ca^2+^] is known to cause apoptosis and/or autophagy [55,56,57]. Since no Ca^2+^ was added to the cell culture medium, the observed increase in [Ca^2+^] indicates mobilization of intracellular calcium pools. Other signs of neurotoxicity of HPU to SH-SY5Y cells await to be evaluated including measurements of pro-inflammatory cytokines. Furthermore, in this experiment, shorter incubation times of cells with HPU should be tested.

The BV-2 cell line exhibits phenotype and functional properties of activated microglia [58]—an essential condition for the immune response in the CNS that the activated microglia are associated with. As discussed in the previous paragraphs, both an increase and a decrease in the concentration of cytosolic Ca^2+^ can be harmful and consequently neurotoxic to cells. Interestingly, in microglial BV-2 cells, HPU did not promote an increase in the levels of intracellular Ca^2+^ (data not shown). The reason for the contrasting responses of these two cell types to HPU remains unknown. It is worth mentioning that these same contrasting responses in the levels of Ca^2+^ were seen for SH-SY5Y and BV-2 cells treated with *Proteus mirabilis* urease, another bacterial urease with neurotoxic properties [59]. 

Overexpression of pro-inflammatory cytokine IL-1β produced by microglia and astrocytes close to Aβ plaques are recurrent in the brain affected by AD, as well as in animal models of AD [60,61]. IL-1β production is dependent on MAPK activation and NF-κB signaling cascade, and studies report that overexpressed IL-1β increases tau phosphorylation and neurofibrillary tangles formation, due to GSK-3β activation [62]. It has been shown that HPU, especially the B subunit, activates the NLRP3 inflammasome, in a TLR2 dependent manner, which is the first step for IL-1β synthesis [18,63]. Here, we report an increase in the production of IL-1β by BV-2 cells primed with HPU (300 nM). Brain homogenates from the 7-day experiment showed an increase in Iba1 expression, indicating an activation status. BV-2 cells also had a significant reduction in viability when treated with HPU. In the 300 nM dose of HPU, viability was reduced by more than 60% (Figure 2). LPS was used as a positive control as it is known to activate BV-2 cells, turning these cells into an inflammatory phenotype, inducing the synthesis of pro-inflammatory markers and reducing BV-2 viability [64].

The neurotoxicity of purified HPU administered to rats intraperitoneally was first described by Baik and colleagues in 2005 [65], who observed hypothermia, convulsion and death of the animals. It should be noted that these same effects occur in rats and mice injected with CNTX, a highly neurotoxic urease isolated from the *Canavalia ensiformis* plant, and these events are independent of the protein’s enzymatic activity [66,67]. However, these symptoms of HPU’s neurotoxicity do not allow establishing, from epidemiological data, that there is a correlation between *H. pylori* infection with AD tauopathy. Mondragón-Rodríguez et al., 2014 [40] reported that phosphorylation in tau’s carboxy terminal region could be related to early AD-related tau phosphorylation events, occurring before the classic appearance of neurofibrillary tangles, one of the hallmarks of the disease. Among tau’s phosphorylation sites, some are located in the proline-rich region (P-region) (residues 172–251) and in the carboxy terminal region (C-region) (residues 368–441) [45]. In this work, we found increased phosphorylation of tau in HPU-treated rats in two sites located in the P-region (Thr205 and Ser199) and one site in the C-region (Ser396). Our findings corroborate previous epidemiological and animal model studies, which described the correlation between *H. pylori* infection and AD [1], and point to a potential role of HPU in this correlation.

In the experimental conditions tested, the animals received a total of 35 μg of HPU over 7 days. Assuming that the total amount of HPU administered was evenly distributed in the body of a 150 g rat, of which 70% is water, the HPU “concentration” in the animal would be approximately 0.33 µg/mL. Under our conditions, the antibodies against either subunit of HPU would still be able to detect the presence of the protein in the brain homogenates in concentrations as low as 0.116 µg/mL of urease. A possible explanation for the negative result shown in Figure 6 is that the Western blot test was not sensitive enough to detect smaller amounts of HPU, if present in the hippocampal homogenates. Despite the negative result, it is still premature to reach the conclusion that HPU administered intraperitoneally could not cross the BBB. It can be argued that even if there was an opening of the BBB, the enormous size of the HPU molecule, with a molecular mass of 1.1 MDa would pose difficulties to its entrance into the CNS. In the article by Baik et al., in 2005 [65], a 20-fold increase in serum ammonia levels was measured in animals treated intraperitoneally with the *H. pylori* filtrate, reflecting the enzymatic action of HPU. It is known that hyperammonemia is a condition that can lead to changes in the blood–brain barrier in humans. Thus, the additive effect of hyperammonemia consequent to HPU’s enzymatic activity and its direct effect on endothelial cells, increasing the paracellular permeability [34], would eventually allow the entry of circulating HPU into the CNS [68,69,70]. On the other hand, it is well known that some proteins, such as tetanus and botulinum neurotoxins, and also viruses, bacteria and protozoans, are actively transported from peripheral sites toward the CNS. Furthermore, the BBB is heterogenous, and some regions, such as around the circumventricular organs, are more permeable due to the presence of fenestrated microvessels and discontinuous tight junctions [71,72].

Extracellular vesicles play an important role in the physiology of the CNS since they act not only in the signal transmission between neurons but as well as in the communication between the CNS and all peripheral systems. As particles on the nanometer scale, they can cross the blood–brain barrier [73]. Microvesicles produced by *H. pylori* carrying CagA were immunolocalized in proximity to occlusive junctions of epithelial cells, from where they are internalized [74]. The presence of HPU in *H. pylori* microvesicles was characterized by mass spectrometry [38]. Moreover, CD46, which is a receptor for HPU [75], is known to play an important role in the uptake of exosomes across the human BBB endothelial cells [76]. Thus, it could serve as a facilitator for HPU entrance into the CNS, either of the free circulating protein or HPU contained in microvesicles.

Finally, it should be considered that a low, but chronic, increase in serum ammonia levels during *H. pylori* infection, which can persist asymptomatic for decades, could be the factor that triggers the hyperphosphorylation of tau as observed here. There are metabolomic studies that identified hyperammonemia associated with animal models of AD [68,77]. In view of these considerations, further studies are still needed to reach a conclusion on the possibility of HPU crossing the BBB, whether of the free protein in the circulation and/or carried by microvesicles produced by *H. pylori*. More sensitive detection methods as well as the search for HPU in other parts of the CNS are underway aiming to elucidate the fate of the protein given by the intraperitoneal route.

Urease-activated signaling routes that may interfere with brain tau phosphorylation should be further investigated. Previous data from our group showed that HPU can modulate signaling pathways involving lipoxygenase-derived eicosanoids and alter intracellular calcium levels in rabbit platelets, human neutrophils and human endothelial cells [28,34,78]. Eicosanoids are known to be involved in neuroinflammation [79,80], a condition that also exists in AD [81,82].

It is well known that biochemical hallmarks precede the cognitive decline observed in AD patients [83]. This may explain why we did not observe any behavioral changes in the conditions tested. Neither memory impairment nor anxiety-like behavior was altered in HPU-treated rats. Surprisingly, although the literature reports that LPS induces injury in the CNS [84] and memory loss under experimental conditions similar to ours [85,86,87], no behavioral alterations were seen here for LPS-treated rats. However, other studies did not find an effect of LPS on behavioral analyses, including different memory tests and anxiety-like behavior [88]. The earliest pathophysiological biomarker of AD is the accumulation of Ab peptide [83]. Wang and co-workers reported in 2014 that the 7-day treatment of 30-day-old rats with the *H. pylori* filtrate promoted impairment of spatial learning and memory loss, accompanied by accumulation of the Ab peptide in the animals’ brain [89]. Besides HPU, other bacterial exotoxin(s) in the filtrate may act synergistically with the urease in promoting neuroinflammation and cognitive impairment. *H. pylori* infection lasts for decades, inducing a chronic inflammatory response. Here the 7 days of treatment with HPU was chosen for comparison with the data presented by Wang et al., 2015 [18], with the *H. pylori*’s filtrate. This condition mimics an infection in young rats (30-day-old); however, although biochemical markers of neuroinflammation and hyperphosphorylated tau were present, the 7-day treatment was clearly not enough to produce behavioral alterations. Thus we are now carrying out experiments to assess behavioral alterations and neuroinflammatory biomarkers after much longer schedules of HPU treatments. 

Altogether, the data presented here are an indication that *H. pylori*’s urease is an important virulence factor also for the development of extra gastric diseases, such as Alzheimer’s disease. HPU’s pro-inflammatory activity and activation of the immune system causing neuroinflammation and tau phosphorylation should not be overlooked as significant contributors to the progression of neurodegenerative diseases.

## 4. Materials and Methods

### 4.1. Cell Culture

Cell lines BV-2 (Banco de Células do Rio de Janeiro, Rio de Janeiro, Brazil) kindly donated by Dr. Silvia Farsky, Universidade de São Paulo, Brazil, and SH-SY5Y (ATCC^®^ CRL-2266^TM^) kindly donated by Dr. Fabio Klamt, Universidade Federal do Rio Grande do Sul, Porto Alegre, Brazil, were maintained in RPMI and DMEM/F12 medium (Invitrogen, Thermo Fisher, Waltham, MA, USA), respectively, supplemented with 10% fetal bovine serum (FBS, HyClone^TM^, GE Healthcare Life 91 Sciences, Logan, UT, USA), 1% Pen/Strep (Invitrogen, Waltham, MA, USA) at 37 °C under 5% CO_2_ humidified atmosphere.

### 4.2. HPU Purification

Recombinant *Helicobacter pylori* urease (HPU) was produced by heterologous expression in *Escherichia coli* BL21 (DE3)-RIL transformed with a PGEM-T-easy (Promega, Madison, WI, USA) plasmid carrying the whole urease operon (kindly provided by Dr. Barbara Zambelli, Universitá di Bologna, Italy). HPU was purified from bacterial extracts according to Wassermann et al., 2010 [28], with small modifications introduced in Scopel-Guerra et al., 2017 [29]. Protein purity was verified by SDS-PAGE (Figure S1).

### 4.3. Protein Determination

The protein content of samples was determined by absorbance at 280 nm, or by the Coomassie dye binding method.

### 4.4. Urease Activity

The urea hydrolyzing activity of HPU was measured by the alkaline nitroprussiate method [30] using ammonium sulfate as a reference, to follow the purification of the holoenzyme. 

### 4.5. Rat In Vivo Assays

Twenty-four male 30-day-old Wistar rats, housed at 22 + 3 °C with a 12/12-h light/dark cycle, were used in the bioassays. Considering that *H. pylori* infection persists lifelong, we selected healthy young animals to simulate a chronic infection and to investigate HPU’s neurotoxic effects in early life stages. Animals were separated into three groups (*n* = 4), and experiments were performed three times, in the same conditions. Control animals received sterile saline intraperitoneal (i.p.,) injections. Treated animals received daily i.p., injections of 5 µg HPU, or 1 mg/kg LPS, for 7 days. Injections of 50 µL were administered in the lower abdomen quadrant, intercalating left and right sides. On the 8th day, animals were anesthetized with a ketamine 5%, xylazine 2% solution and euthanized by decapitation, using a small rodent guillotine. 

For the behavioral experiment, 30 male 30-day-old Wistar rats, housed at 22 + 3 °C with a 12/12-h light/dark cycle, were used. Animals were separated into three groups (*n* = 10). Control animals received sterile saline intraperitoneal (i.p.,) injections. Treated animals received daily i.p., injections of 5 µg HPU or 1 mg/kg LPS, for 7 consecutive days. Injections of 100 µL were administered in the lower abdomen quadrant, intercalating left and right sides. On the 8th day, animals were submitted to the behavioral tests described in Section 4.12. After the behavioral tests, animals were anesthetized with a ketamine 5%, xylazine 2% solution and euthanized by decapitation, using a small rodent guillotine. 

### 4.6. Preparation of Brain Homogenates

Rat brains were dissected, the hemispheres were separated and a medial fraction of brain tissue containing the hippocampus and the temporal lobe was collected. The tissues were homogenized in lysis buffer (20 mM Tris-HCl, 1% NP-40, 1 mM EDTA, 1 mM EGTA, 1 mM PMSF, 1 mM Na_3_VO_4_, protease inhibitor cocktail (Sigma, St. Louis, MO, USA) using a microtube pestle. Tissue homogenates were centrifuged at 12,000× *g* for 10 min, at 4 °C, and stored at −80 °C.

### 4.7. Western Blot Analysis

Brain homogenates were denatured in sample buffer (50 mM Tris-HCl, pH 6.8, 1% SDS, 5% 2-mercaptoethanol, 10% glycerol, 0.001% bromophenol blue) and heated in a dry bath for 5 min. Samples (20 µg total protein) were resolved in SDS-PAGE gels and proteins were transferred to 0.22 µm nitrocellulose membranes (BioRad, Hercules, CA, USA). Molecular mass markers (PageRuler, Thermo-Scientific, Waltham, MA, USA) were run in parallel. Membranes were blocked with PBS-Tween (137 mM NaCl, 2.7 mM KCl, 10 mM Na_2_HPO_4_, 1.8 mM KH_2_PO_4_, 0.1% Tween-20), containing 5% bovine serum albumin (BSA; Sigma) and probed with the following antibodies: mouse anti-Tau46 (Cell Signaling, #4019, 1:1000), rabbit anti-ptau205 (Invitrogen, 44-738G, 1:1000), rabbit anti-ptau396 (Invitrogen, 710298, 1:1000), rabbit anti-ptau199 (Invitrogen, 701054, 1:1000), rabbit anti-Iba1 (Invitrogen, PA5-27436, 1:1000), rabbit anti-GSK-3beta (Sigma-Aldrich, St. Louis, MO, USA, PK1111, 1:1000), rabbit anti-ureβ (Santa Cruz Biotech, Santa Cruz, CA, USA, sc-22742, 1:10,000), rabbit anti-Ureα (Santa Cruz Biotech, Santa Cruz, CA, USA, sc-21016, 1:10,000), rabbit anti-actin (Sigma-Aldrich, St. Louis, MO, USA, A2066, 1:1000). Secondary antibodies (anti-mouse and anti-rabbit, 1:10,000) coupled to horseradish peroxidase were from Jackson ImmunoResearch Laboratories, Inc., West Grove, PA, USA). The protein bands were visualized using a chemiluminescence detection kit (Millipore, Billerica, MA, USA) in a L-Pix Chemi (Loccus, Cotia, Brazil) apparatus. The levels of protein expression were quantified using the software ImageJ and normalized against β-actin as an endogenous control. 

### 4.8. Reactive Oxygen Species (ROS) Measurement

In order to measure intracellular ROS production, carboxy-H2DCFDA (5-(-6)-carboxy2′,7′-difluorodihydrofluorescein diacetate, Thermo-Scientific, Waltham, MA, USA) was used following the protocols described in [59]. Cells (10^6^ cells/well) were seeded in 96-well plates and incubated with the dye (2 mM) for 30 min at 37 °C in the dark, and then stimulated with 50, 100 or 300 nM HPU or 100 µM H_2_O_2_, and ROS production was measured 6 and 24 h after treatment. Fluorescence (λ_ex_ 495 nm/λ_em_ 527 nm) was measured using a Spectramax microplate reader (Molecular Devices, San Jose, CA, USA). Experiments were performed in triplicate. The time dynamics in fluorescence were normalized against the control group df/F0 (%), where F0 represents the fluorescence expressed by the control and df represents the change in fluorescence over time during the cell stimulus.

### 4.9. Intracellular Calcium Measurement

Calcium concentration was measured using the Fluo-4 dye (Invitrogen, Waltham, MA, USA F14201) following the protocol described in [59]. Cells (10^6^ cells/well) were seeded in 96-well plates and incubated with the dye (5 µM) for 45 min at 37 °C in the dark. The dye solution was removed, and cells were washed 3 times with 20 mM phosphate buffer pH 7.5, to remove excess dye. Cells were stimulated with 50, 100 or 300 nM HPU or 35 mM KCl and calcium concentration was measured 6 and 24 h after treatment. Fluorescence (λ_ex_ 488 nm/λ_em_ 530 nm) was measured using a Spectramax microplate reader (Molecular Devices, CA, USA). Experiments were performed in triplicate. The time dynamics in fluorescence were normalized against the control group df/F0 (%), where F0 represents the fluorescence expressed by the control and df represents the change in fluorescence over time during the cell stimulus. 

### 4.10. Cytokine Measurement

BV-2 cells were cultured in 96-well plates and incubated with 10, 50, 100 or 300 nM HPU. After 6 h, supernatants were collected and stored at −80 °C. ELISA kits were used to detect the expression of IL-1β (88–7013-22) and TNF-α (88–7324-22) according to the manufacturer’s instructions (Thermo-Scientific, Waltham, CA, USA).

### 4.11. Cell Viability

The viability of BV-2 cells after HPU treatment was evaluated using the Thiazolyl Blue Tetrazolium Blue assay (MTT; M2128; Sigma-Aldrich, St. Louis, MO, USA), according to the manufacturer’s instructions. Cells were treated for 24 h with 10, 50, 100 or 300 nM HPU. After treatment, cells were incubated with MTT solution (5 mg/mL) for 4 h at 37 °C. The solution was removed and 100 µL DMSO was added to solubilize the precipitates. The plates were read at 570 nm in an M2 spectrophotometer (Molecular Devices, San Jose, CA, USA). The data is expressed as a percentage compared to control.

### 4.12. Behavioral Tests

#### 4.12.1. Object Recognition Task

The method described by Ennaceur and Delacour, 1988 [90], was followed. An open field arena (60 × 40 × 50 cm) was placed in a dimly illuminated room. The objects to be discriminated were made of steel or glass and were carefully cleaned with 70% ethanol after each test to ensure the absence of olfactory cues. Exploration was defined as sniffing or touching the object with the nose and/or forepaws. The animals were habituated to the arena, exploring it for 4 consecutive days, 20 min per day, 6 days before the test. On the day before the test, the animals were placed in the open field arena with two identical objects (A and A) and explored for 5 min (training). On the day of the test, the animals were placed in the same apparatus for 5 min with a familiar and a novel object (A and B). In order to quantify object recognition memory, discrimination indexes (DI) were calculated as follows: time spent exploring the novel object subtracted the time spent exploring the familiar object, divided by the total exploration time spent exploring both objects (tnov − tfam)/(tnov + tfam).

#### 4.12.2. Elevated Plus Maze

The method described in Furini et al., 2010 [91], was applied. Enhanced anxiety was evaluated using the elevated plus maze test (EPM). The EPM is formed by two open arms and two closed arms of the same size, united to a central square (10 cm^2^), elevated 45 cm from the floor. Two opposite arms had walls (10 cm high). Each arm was 40 cm long and 10 cm wide. The animals were placed in the center and had 5 min for free exploration. For measurement purposes, we counted the number of entries in each arm and the time spent in open arms. 

### 4.13. Statistical Analysis

All results are expressed as average ± standard error of the mean (SEM) of at least three experiments, unless otherwise stated. The statistical significance of the differences between two groups was assessed using the unpaired Student’s *t*-test and for multiple comparisons, a one-way analysis of variance (ANOVA) was performed followed by Tukey’s post hoc test. Statistical significance was set at *p*-value ≤ 0.05. GraphPad Prism 6 software (San Diego, CA, USA) was used to perform statistical analysis.

## Supplementary Materials

The following are available online at https://www.mdpi.com/article/10.3390/ijms23063091/s1.
