# Supplementary figures and images for: Helicobacter pylori Urease: Potential Contributions to Alzheimer’s Disease
Source: Int J Mol Sci. 2022 Mar 13;23(6):3091. doi: 10.3390/ijms23063091 (PMC8949269; doi:10.3390/ijms23063091)

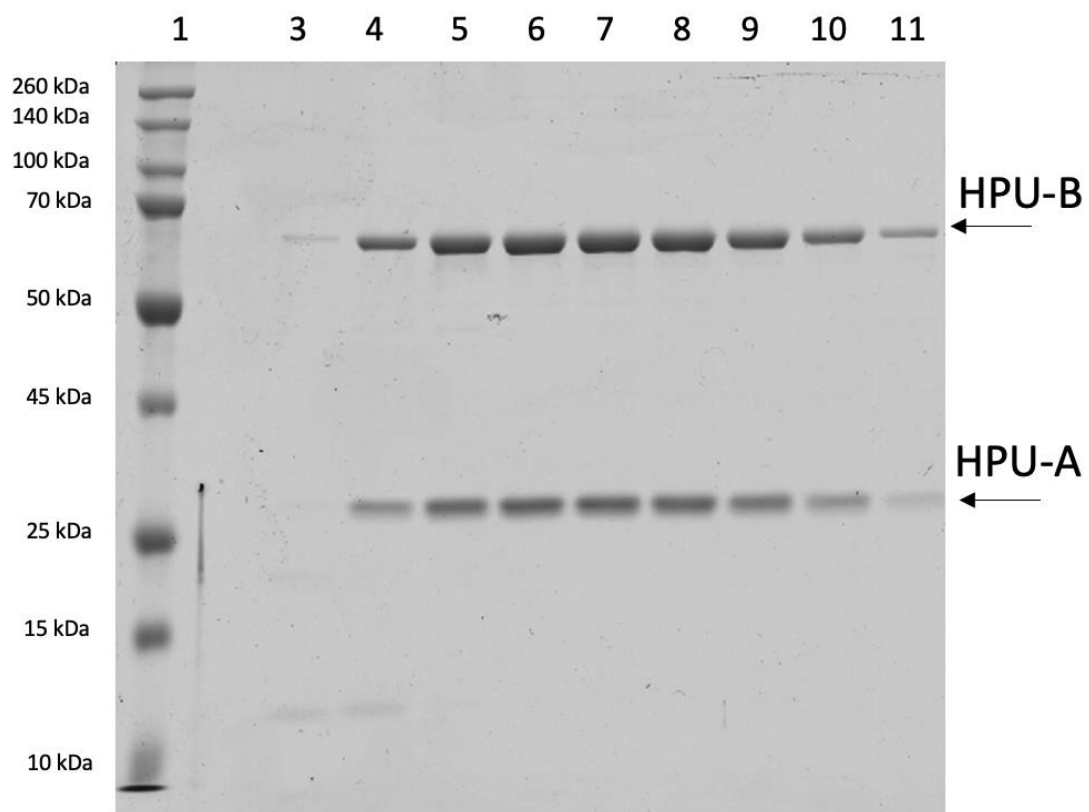

**Figure S1:** Purified recombinant *H. pylori* urease.

Supplement: Supplementary file 1 [file ijms-23-03091-s001.zip › ijms-1538683-supplementary.pdf]
